# Incidence and endoscopic characteristics of acute laryngeal lesions in children undergoing endotracheal intubation^☆^

**DOI:** 10.1016/j.bjorl.2015.09.012

**Published:** 2016-01-07

**Authors:** Eliandra da Silveira de Lima, Maíra Alves Braga de Oliveira, Carolina Rocha Barone, Kharina Mayara Moreira Dias, Samanta Daiana de Rossi, Claudia Schweiger, Denise Manica, Larissa Valency Enéas, Catia de Souza Saleh Netto, Gabriel Kuhl, Paulo Roberto Antonacci Carvalho, Paulo Jose Cauduro Marostica

**Affiliations:** a Universidade Federal do Rio Grande do Sul (UFRGS), Porto Alegre, RS, Brazil; b Hospital de Clínicas de Porto Alegre (HCPA), Departamento de Otorrinolaringologia, Porto Alegre, RS, Brazil; c Universidade Federal do Rio Grande do Sul (UFRGS), Programa de Pós Graduação em Saúde da Criança e do Adolescente, Porto Alegre, RS, Brazil; d Hospital de Clínicas de Porto Alegre (HCPA), Unidade de Terapia Intensiva Pediátrica, Porto Alegre, RS, Brazil; e Hospital de Clínicas de Porto Alegre (HCPA), Unidade de Pneumologia Pediátrica, Porto Alegre, RS, Brazil

**Keywords:** Intubation, Laryngeal diseases, Laryngoscopy, Artificial respiration, Intubação, Doenças da laringe, Laringoscopia, Respiração artificial

## Abstract

**Introduction:**

Acute laryngeal lesions after intubation appear to be precursors of chronic lesions.

**Objective:**

To describe the incidence and type of acute laryngeal lesions after extubation in a pediatric intensive care unit (PICU).

**Methods:**

A cohort study involving children from birth to <5 years, submitted to intubation for more than 24 h in the PICU of an university hospital. In the first eight hours after extubation, a flexible fiberoptic laryngoscopy (FFL) was performed at the bedside. Those with moderate to severe abnormalities underwent a second examination seven to ten days later.

**Results:**

177 patients were included, with a median age of 2.46 months. The mean intubation time was 8.19 days. Seventy-three (41.2%) patients had moderate or severe alterations at the FFL, with the remaining showing only minor alterations or normal results. During follow-up, 16 children from the group with moderate to severe lesions developed subglottic stenosis. One patient from the normal FFL group had subglottic stenosis, resulting in an incidence of 9.6% of chronic lesions.

**Conclusion:**

Most children in the study developed mild acute laryngeal lesions caused by endotracheal intubation, which improved in a few days after extubation.

## Resumo

**Introdução:**

As lesões laríngeas agudas após a intubação parecem ser precursoras das lesões crônicas.

**Objetivo:**

Descrever a incidência e o tipo de lesões laríngeas agudas após extubação em Unidade de Terapia Intensiva Pediátrica (UTIP).

**Método:**

Estudo de coorte envolvendo crianças de 0 a 5 anos incompletos, com intubação por mais de 24 horas na UTIP de um hospital universitário. Nas primeiras 8 horas após extubação, uma nasofibrolaringoscopia à beira do leito foi realizada. Aqueles com anormalidades moderadas a graves foram submetidos a novo exame entre 7–10 dias após.

**Resultados:**

177 pacientes foram incluídos, com idade mediana de 2,46 meses. O tempo médio de intubação foi de 8,19 dias. Setenta e três (41,2%) pacientes apresentaram alterações moderadas ou graves à laringoscopia, o restante mostrando apenas alterações leves ou exame normal. Durante o acompanhamento, 16 crianças do grupo lesões moderada a grave desenvolveram estenose subglótica. Um paciente do grupo laringoscopia normal teve estenose subglótica, somando-se uma incidência de 9,6% de lesões crônicas.

**Conclusão:**

A maioria das crianças do estudo desenvolveu lesões laríngeas agudas leves decorrentes da intubação endotraqueal, com melhora em alguns dias após a extubação.

## Introduction

It is estimated that one in three patients admitted to a pediatric intensive care unit (PICU) will require endotracheal intubation for an average of five days.^1^

The endotracheal tube exerts pressure on the mucosa of the posterior aspect of the larynx; the resulting ischemia seems to be the starting point for the development of post-intubation acute laryngeal lesions. The lesions occur at the points of greatest contact with the tube: medial surface of the arytenoid cartilage, medial portion of cricoarytenoid joint and vocal process, posterior glottis in the interarytenoid region, and subglottis involving the inner surface of the cricoid cartilage, usually the posterior portion.^2^, ^3^, ^4^

There are several classifications for acute lesions secondary to intubation. According to Lindholm, the lesions can be classified from grades I to IV, depending on severity.^5^ According to Benjamin, acute laryngeal lesions are divided into five groups: early nonspecific alterations, edema, granulation tissue, ulceration, and miscellaneous.^3^ Fan et al. classified the findings as normal or mild (interarytenoid region ulceration, vocal fold granuloma), moderate (pseudomembranes, bulky granulomas) and severe lesions (subglottic stenosis, subglottic membrane, tracheal stenosis, vocal fold paralysis).^6^ Colice et al. classified the findings as normal, mild (erythema or mucosal ulceration without lumen size reduction during inspiration), moderate (erythema, ulceration, and mucosal edema, reducing laryngeal lumen during inspiration) and severe lesions (erythema, ulceration, and mucosal edema reducing laryngeal lumen by more than 50% during inspiration).^7^ This lack of homogeneity in classifications makes it difficult to compare the studies.

The incidence of subglottic stenosis (SGS) in the PICU of the university hospital where this study was performed was estimated at 11.3%.^8^ Due to the fact that severe chronic lesions generally result from the evolution of acute lesions,^9^ it is of utmost importance to know the epidemiology of the latter, in order to generate data for the prevention of severe laryngeal lesions.

Therefore, this study aimed to describe the incidence and type of acute laryngeal lesions after extubation in the PICU.

## Methods

This cross-sectional study involved a cohort of children, aged from birth to <5 years, admitted to the PICU of a university hospital between November of 2005 and November of 2012 who were submitted to endotracheal intubation for more than 24 h and whose parents or guardians authorized the inclusion in the study. Exclusion criteria included patients with previous history of stridor or known laryngeal disease, history of endotracheal intubation, presence or history of tracheotomy, and patients considered to be critically ill by the care team.

In the first eight hours after extubation a flexible fiberoptic laryngoscopy (FFL) was performed in the PICU with the patient in bed and without sedation. The fiberoptic laryngoscope was introduced only to the supraglottic region, aiming to obtain images of the supraglottic, glottic, and subglottic regions. The glottic level was not surpassed in any patient, to avoid triggering a laryngospasm. The recorded movies were evaluated by a researcher (GK) blinded to the other patients’ data and experienced in FFL.

The definitions used to classify lesions by FNL after extubation were: hyperemia – classified as mild when affecting up to one-third of the structure, moderate when affecting more than two-thirds, and intense when affecting the entire structure; edema – classified as mild when affecting up to one-third of the structure, moderate when more than two-thirds were affected, and intense when affecting the entire structure; vocal fold bleeding; immobility – defined as the absence of hemilarynx movement and classified according to the laterality (right, left, and bilateral); ulceration – discontinuity of the mucosa that overlays the larynx; and granulation – emergence of abnormal tissue of granulomatous appearance. Both ulcerations and granulations were classified according to location: if in the glottis, as unilateral (when affecting one vocal process of the arytenoid), bilateral (when affecting both vocal processes of the arytenoid), or interarytenoid (when occupying the posterior wall between the vocal processes); if in the subglottic region, as partial (less than 360° of the lumen) or total (affecting 360° of the lumen). Furthermore, the presence of obstructive (if it prevented the visualization of the glottis) or non-obstructive laryngomalacia was also described.

Therefore, the lesions classified as mild alterations included: hyperemia, edema, vocal fold hemorrhage, and non-obstructive laryngomalacia; as moderate: obstructive laryngomalacia, unilateral or bilateral glottal ulceration, arytenoid granulation, and partial subglottic ulceration; and as severe: vocal fold immobility, interarytenoid ulceration, interarytenoid granulation, total subglottic ulceration, and subglottic granulation (Table 1). Laryngomalacia was included as acute laryngeal lesion because, if obstructive, it would not allow the visualization of the glottic and subglottic structures. Hence, these patients were followed as possible cases of acute laryngeal lesion, and their endoscopy was repeated between seven and ten days after the first assessment, as explained subsequently.

**Table 1:** Classification of FFL findings as mild, moderate, or severe, according to the anatomical location.

Anatomical location	Classification		
	Mild	Moderate	Severe
Supraglottis	Edema	Obstructive LM	
	Hyperemia		
	Non-obstructive LM		

Glottis	Edema	Unilateral or bilateral ulceration	Immobility
			Interarytenoid ulceration
	Hyperemia	Arytenoid granulation	Interarytenoid granulation

Subglottis	Edema	Partial ulceration	Total ulceration
	Hyperemia		Granulation

FFL, flexible fiberoptic laryngoscopy; LM, laryngomalacia.

During the period when they remained in the PICU, the children were monitored daily by the researchers, collecting data such as mobilization of endotracheal tube by the assistant staff, number of reintubations, need for an increase in basal dose of sedation, or use of extra doses of sedation, which are the additional doses of sedation in addition to that regularly prescribed.

**Figure 1:**
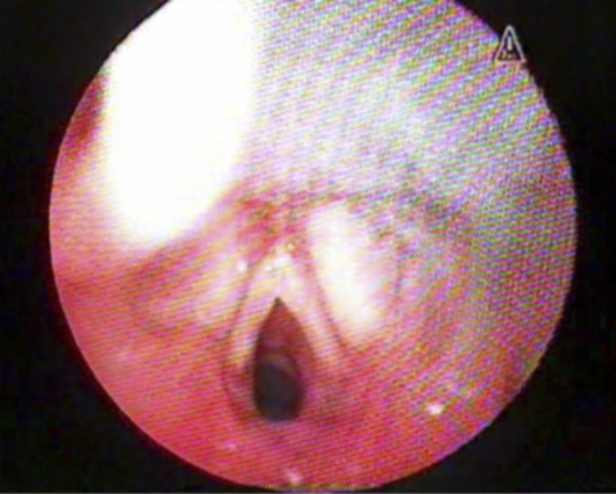
Flexible fiberoptic laryngoscopy after extubation: normal subglottis; mild posterior glottic edema.

Patients with normal results or slight alterations (Fig. 1) had a follow up consultation in the Pediatric Laryngology Outpatient Clinic of the Hospital Otorhinolaryngology Service one month after discharge. Those who remained laryngologically asymptomatic (absence of stridor, laryngitis, dysphonia, or swallowing disorders) were followed by monthly telephone contact after the outpatient assessment, up to a total of 12 months. Otherwise, they underwent airway endoscopy under general anesthesia.

**Figure 2:**
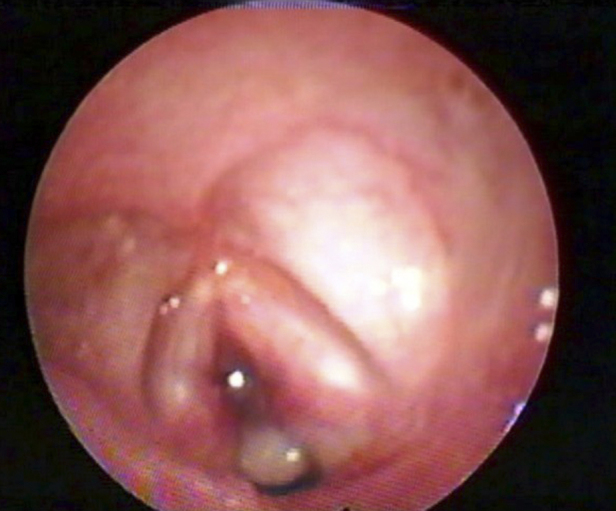
Flexible fiberoptic laryngoscopy after extubation: extensive anterior subglottic granulation.

Patients with moderate to severe alterations (Fig. 2) underwent FFL a second time between seven and ten days after extubation. When that examination revealed normal results, the patients were followed in the same way as the patients with early findings classified as normal or mild. When lesions were identified in this second examination, the patients were referred for airway endoscopy under general anesthesia.

This study was approved by the Research Ethics Committee of the hospital under No. 05-266 and the parents or legal guardians signed an informed consent prior to study enrollment.

The variables are shown as proportions according to the assessed categories.

## Results

There were 177 patients included, with a median age of 2.46 months, of whom 59.9% were males. The most commonly used route of intubation was the orotracheal route, whereas the nasotracheal intubation was performed in only one patient. An endotracheal tube with cuff was used in 19.4% of patients. The mean intubation time was 8.19 days. The causes of intubation were bronchiolitis (63.3%), pneumonia (13.6%), meningitis (5.6%), respiratory dysfunction (5.1%), asthma (2.3%), and other diagnoses (10.2%).

The FFL performed immediately after the extubation showed that 104 (58.8%) patients had normal results or only mild alterations, whereas 73 (41.2%) patients had moderate or severe alterations in addition to possible minor ones. Overall, the most frequently found mild alteration was edema, of supraglottic location in 138 patients (78%), followed by posterior glottic edema in 127 patients (72.2%), vocal fold edema in 85 patients (48%), and subglottic edema in 48 patients (27.8%). Hyperemia was the second most common mild alteration, found in the supraglottic location in 122 patients (69%), in the posterior glottis in 106 patients (59.8%), and in the subglottic region in 76 patients (43.7%). Other acute alterations were non-obstructive laryngomalacia (22%) and vocal fold bleeding (5.1%).

The most common moderate alterations were arytenoid granulation in 36 patients (20.5%) and unilateral or bilateral glottal ulceration in 29 patients (16.6%). Other moderate changes were obstructive laryngomalacia (1.7%) and partial subglottic ulceration (1.7%).

The most frequent severe alteration was subglottic granulation, identified in 36 patients (21%). The other severe alterations were complete subglottic ulceration (4%), interarytenoid granulation (3.4%), vocal fold immobility (1.7%), and interarytenoid ulceration (0.6%).

It was not possible to visualize the subglottic region in nine patients, due to the presence of glottic granulation in seven cases and due to obstructive laryngomalacia in two cases. These patients underwent a new assessment after seven to ten days. At the new assessment, only one patient with glottic granulation developed subglottic stenosis. In this case, the patient underwent laryngoscopy under sedation.

During the follow-up after extubation, 16 patients in the group with moderate or severe alterations developed subglottic stenosis. Only one patient with normal FFL or with mild alteration developed subglottic stenosis, with a total incidence of chronic lesions of 9.6%.

## Discussion

In the study by Smith et al., with data obtained from a PICU between December of 2005 and November of 2006 in patients from the present line of research, the overall prevalence of alterations in the FFL in children post-extubation was 92.68%; of this total, 51.22% were mild lesions; 14.63% were classified as moderate, and 26.83% as severe. The most frequent moderate to severe lesions were posterior glottic granulation, subglottic granulation, and posterior glottic ulceration.^10^ At the analysis performed in the present study, with data collected during seven years, the percentage of lesions and the degree of severity were similar to the results obtained by Smith et al.

In the prospective study by Cordeiro et al., carried out in patients from the neonatal ICU and PICU of the Hospital da Universidade de São Paulo, the overall prevalence of post-intubation laryngeal lesions in children was 89.9%. Of this total, the mild lesions accounted for 54.8%; moderate for 24.2%; and severe, for 10.7%. The most frequently found moderate lesion was vocal fold edema, followed by ulceration. The most frequently found severe lesions were fibrous nodules on the vocal fold, followed by synechias.^11^ The inclusion of newborns in the study and the use of another lesion classification may explain the different results compared to this study, especially for the severe lesions.

Post-intubation laryngeal lesions are mostly temporary. However, when present, they significantly impact patients’ health and quality of life and may prolong hospitalization. When located in the anterior portion of the glottis, the main presenting symptom is dysphonia or hoarse cry. The lesions located in the posterior glottic and subglottic regions feature stridor and respiratory dysfunction as the main symptoms. Some lesions such as granulomas can also cause foreign body sensation, cough, clearing of the throat, and laryngeal pain.^3^, ^12^ Additionally, acute laryngeal lesions may be precursors of chronic lesions and should be closely monitored.

## Conclusion

This study shows that most children have acute lesions resulting from endotracheal intubation, but most of these lesions are mild and improve within a few days after extubation.

## Conflicts of interest

The authors declare no conflicts of interest.
